# Self-Consistency Congruence and Smartphone Addiction in Adolescents: The Mediating Role of Subjective Well-Being and the Moderating Role of Gender

**DOI:** 10.3389/fpsyg.2021.766392

**Published:** 2021-12-06

**Authors:** Yang Li, Xiaoqing Ma, Chun Li, Chuanhua Gu

**Affiliations:** ^1^Key Laboratory of Adolescent Cyberpsychology and Behavior (Central China Normal University), Ministry of Education, Hubei Province Key Laboratory of Human Development and Mental Health, Wuhan, China; ^2^Center of Mental Health Education, Nanchang University, Nanchang, China; ^3^Central China Normal University Branch, Collaborative Innovation Center of Assessment Toward Basic Education Quality, Wuhan, China

**Keywords:** subjective well-being, mobile phone addiction, gender, mediating effect, self-consistency congruence

## Abstract

Adolescent smartphone addiction has increasingly attracted the attention of scholars because of the widespread use of internet technology in educational environments. In addition, previous studies have found that there is a complex relationship between smartphone addiction and self-consistency congruence, and subjective well-being. This research was conducted to examine whether subjective well-being would mediate the relation between self-consistency congruence and adolescent smartphone addiction, and whether gender would moderate the mediating process. A total of 1,011 Chinese adolescents completed self-report questionnaires measuring self-consistency congruence, subjective well-being, and smartphone addiction. Self-consistency congruence was shown to be a significant predictor of smartphone addiction. Furthermore, subjective well-being partially mediated the association between self-consistency congruence and adolescent smartphone addiction. Gender could moderate the mediating process; as compared with boys, girls’ self-consistency congruence and subjective well-being are more easily mediated. We envision the findings as being helpful in guiding scholars who are developing interventions to minimize smartphone addiction and its disrupting effects in adolescents.

## Introduction

Smart phones are rapidly being popularized with the continuous development of communication technology. Statistics show that by 2017, the penetration rate of smartphones in the world was as high as 30.90% (Statista, 2018). As one of the developed countries, the penetration rate of smartphones in the United States has reached 64% as early as 2014 (Smith, 2015). The use of smartphones as Internet terminals has changed people’s lifestyles. No matter where they are, we can always see people using smartphones. The widespread use of smartphones has become a worldwide phenomenon (He et al., 2012), and while social networking, shopping, entertainment, and gaming have benefited people, they have also caused damage (Billieux, 2012; Gosling and Mason, 2015). Specifically, inappropriate use of smartphones can reduce individuals’ attention and cognition (Campbell, 2006; Lepp et al., 2014), hinder face-to-face communication (Pierce, 2009), and even lead to mental or physical problems (Beranuy et al., 2009; Thomee et al., 2011). Among all these negative outcomes, smartphone addiction is probably one of the most direct negative outcomes of smartphone use (Takao et al., 2009; Choliz, 2010).

Smartphone addiction is a strong and persistent addictive behavior that desires and depends on the use of smartphones. Once the addict stops using it, it will produce a withdrawal reaction, and this reaction will lead to the addict’s society and mental function is significantly impaired (Lim et al., 2009). Smartphone addiction has been found not only to affect the physical health of individuals, such as by causing neck, shoulder, and back pain, and hearing and vision loss (Jenaro et al., 2007), but also their mental health, such that smartphone-dependent individuals exhibit varying degrees of mental health problems, including somatization, interpersonal sensitivity, hostility, paranoia, psychoticism (Huang et al., 2013), and anxiety and depression (Kasesniemi and Rautiainen, 2002).

Indeed, in recent years, researchers have made smartphone addiction a focus of study, concentrating on the mechanisms of smartphone addiction (Billieux, 2012), its causes (Bianchi and Phillips, 2005; Toda et al., 2008; Walsh et al., 2011), the relationship between smartphone addiction and physical health (Lonn et al., 2005), the degree of impact of smartphone addiction on individual mental health (Çagan et al., 2014), the relationship between smartphone addiction and academic performance (Lepp et al., 2014) smartphone addiction and behavioral styles (Kamibeppu and Sugiura, 2005; Wang et al., 2014), and the relationship between smartphone addiction and leisure styles (Lepp et al., 2015).

Researchers have found that the study of smartphone addiction has mostly focused on college students (Liu et al., 2017), however, adolescents also have a high rate of smartphone use, and the incidence of smartphone addiction is increasing among this population (He et al., 2012). With the popularity of smartphones and the internet, smartphones have become the most important tool for adolescents to conduct online activities. In addition, adolescence is a period of rapid physical and mental development, and self-development is the core content of psychological development at this stage (Carter, 1969). Studies have found that self-esteem (Kim and Koh, 2018), self-management (Gökçearslan et al., 2016), self-efficacy (Chiu, 2014), self-control (Yu and Son, 2016), self-recovery (Hwang and Kwon, 2016) and other self-related variables are all related to smartphone addiction. It is worth noting that self-consistency congruence is also an important variable of self (Rogers, 1959). The research of Li (2010), Zhang and Lin (2014) shows that self-consistency congruence is negatively correlated with college students’ smartphone addiction, that is, the greater the degree of self-consistency congruence low, the higher the degree of smartphone addiction. Based on the cognitive-behavioral model of pathological Internet use by Davis (2001), negative cognition of oneself is an important factor leading to addiction. Teenagers whose self and experience are not in harmony will experience a lot of conflicts, contradictions, and constraints, and smartphones can provide them with an anonymous and stimulating space to release their inner tension and ease their negative self-experience, which leads to the success of smartphones increased addiction.

However, current research focuses on exploring the direct relationship between smartphone addiction and self-consistency congruence (Liu and Yao, 2006), and both the mediating effect (how self-consistency congruence affects smartphone addiction) and the moderating effect (under what conditions the effect of smartphone addiction on self-consistency congruence is stronger or weaker) between smartphone addiction and self-consistency congruence deserve in-depth analysis. Therefore, this study will explore the effects of smartphone addiction on self-consistency congruence and its mechanisms of action in an adolescent population.

### Smartphone Addiction

Previous studies have shown that scholars, both nationally and internationally, have mainly emphasized the psychological and behavioral characteristics of smartphone addiction. For example, Lee (2002) reported that 73% of respondents described having negative feelings of irritability and restlessness when they could not use their smartphones or had to turn them off. Bianchi and Phillips (2005) also showed that smartphone addicts had difficulty in controlling their smartphone use time and were distracted by smartphone use behaviors when completing other tasks, compared to non-smartphone addicts; they also generated more interpersonal conflicts than non-smartphone addicts. In his 2009 report, Shi (2009) defined smartphone addiction as a state of obsession in which an individual’s physiological, psychological, and social functioning is significantly impaired as a result of uncontrolled smartphone use. Furthermore, researchers generally agree that smartphone addiction does not involve any substance intake and belongs to a new type of compulsive dependence behavior, namely, “behavioral addiction” (Bianchi and Phillips, 2005; Billieux et al., 2008).

Nowadays, smartphones are ubiquitous, and smartphone use among adolescents has increased dramatically, especially in China. As the largest cellphone market in the world, China had 9.86 billion cellphone users at the end of 2021 (CNNIC, 2021), and smartphones are a double-edged sword for young people. On the one hand, the use of smartphones helps young people increase the frequency of social interactions, improve their relationships, and make new friends (Park and Lee, 2012), meanwhile it has also increased the ways for young people to obtain knowledge and information (Samaha and Hawi, 2016). On the other hand, inappropriate use of smartphones can have negative effects on young people. Studies have noted the importance of smartphone addiction on adolescent development, mostly examining the role of micro and mesosystems such as peers, family, or school (Li C. et al., 2016). Some studies have also examined individual factors influenced by smartphone addiction, and domestic and international studies have shown that personality and emotions have a certain influence on smartphone addiction, with both self-consistency congruence and subjective well-being among the influencing factors (Liu and Yao, 2006).

### Self-Consistency Congruence

Self-consistency congruence is one of the most important concepts of C. R. Rogers’s personality theory. According to Rogers, the self is the perception and meaning of the individual’s phenomenal domain (including the individual’s perception of the outside world and of himself) in relation to himself (Wang, 1994). At the same time, the individual has the function of maintaining coherence between various self-perceptions and coordinating the relationship between self and experience, and “most of the actions taken by the individual are consistent with his or her self-concept” (Tian and Zhao, 2006). Thus, self-consistency congruence refers to the coherence within the self and the coordination between the self and experience (Wang, 1994). If an individual experiences a gap between self and experience, internal tension and disturbance, a state of “disharmony,” results (Wang, 1994). In order to maintain their self-concept, individuals resort to a variety of defensive responses and thus provide the basis for psychological disorders to emerge (Wang, 1994).

Self-consistency congruence requires communication with others. Some studies have shown a close relationship between self-consistency congruence and mental health (Liu and Yao, 2006). Previous studies have shown a negative correlation between self-consistency congruence and cellphone addiction among college students; in other words, the lower the degree of self-consistency congruence, the higher the degree of cellphone addiction (Li, 2010). At the same time, self-consistency congruence also affects individuals’ subjective well-being, and differences in self-consistency congruence cause changes in well-being (Yue et al., 2006). A study involving college students found a positive correlation between self-consistency congruence and subjective well-being (Pan, 2006).

Studies indicate that smartphone addiction among college students is negatively correlated with self-consistency congruence, while self-consistency congruence is positively correlated with subjective well-being.

The above research results suggest to a certain extent the close relationship between self-consistency congruence and smartphone addiction. However, the intermediary and regulatory mechanisms that can explain this association are largely unexplored. In order to fill this gap, this study constructed a moderated mediation model to examine the mediating role of subjective well-being between self-consistency congruence and smartphone addiction, and the mediating role of gender between the two.

### The Mediating Role of Subjective Well-Being

Subjective well-being (SWB) is the overall evaluation of an individual’s quality of life based on subjective standards (Diener, 1984; Myers and Diener, 1995; Diener, 2000). In Diener’s (1984) original formulation, subjective well-being includes three main components: Life Satisfaction (LS), positive affect (PA) and negative affect (NA). A high level of subjective well-being is regarded as an important personal and social goal by researchers and the general public (Diener, 2000; Seligman, 2000). Studies have shown that subjective well-being not only contributes to the improvement of adolescents’ academic achievement, but also benefits their mental health development (Padhy et al., 2011; Palomar-Lever and Victorio-Estrada, 2014).

According to Rogers (1959), if a person’s actual experience is close to their perceived ideal state, they are closer to a state of self-consistency congruence (Rogers, 1959). On the contrary, it will be regarded as inconsistency, which will cause the individual to experience negative emotions, maladjustment and psychological distress (Rogers, 1959), and reduce their subjective well-being as a whole. This influence does not end here; it extends to the individual’s network behavior. Based on Bryant and Zillmann’s (1984) Mood Enhancement Hypothesis, individuals determine the time and type of internet use based on their emotional state, and negative emotions increase the probability of problematic internet use (Park et al., 2013). In this process, if individual Internet satisfaction continues to be significantly stronger than reality satisfaction, it is very likely to trigger smartphone addiction (Chiu, 2014; Demirci et al., 2015; Ahn, 2016). Therefore, we can speculate that subjective well-being may be an important mediating variable between self-consistency congruence and smartphone addiction. So far, no research has tested whether subjective well-being plays an intermediary role in the relationship between self-consistency congruence and adolescents’ smartphone addiction. However, there is some preliminary support for this intermediary process in the literature.

First, the state of inconsistency may reduce the individual’s subjective well-being. Some early studies have confirmed that the inconsistency between internal self and external experience is related to various forms of psychological distress (Festinger, 1957). However, subjective well-being is not only the absence of negative emotions (NA); it also includes the existence of positive emotions (PA) and the cognitive factor of life satisfaction (Diener, 1984). Therefore, it is impossible to fully capture the comprehensive relationship between self-consistency congruence and subjective well-being only by studying negative emotions. As research addressed the topic further, Weinstein et al. (2012) started from the opposite perspective and found that self-consistency congruence promotes overall subjective well-being, because the feeling of self-consistency congruence is a vibrant and growth-promoting experience. In that study, self-consistency congruence has a predictive effect on various indicators of subjective well-being. Explained from the perspective of psychoanalysis, when the ideal self is consistent with the situational perception of the self, a state of self-consistency congruence appears and happiness is aroused (Sandler and Rosenblatt, 1962; Joffe and Sandler, 1965). It can be seen that there is an extremely close connection between self-consistency congruence and subjective well-being.

Second, the decrease in subjective well-being may trigger adolescents’ smartphone addiction. Based on a large amount of behavioral evidence, individuals with different levels of happiness have systematic differences in a variety of cognitive, motivational and emotional processing processes (Lyubomirsky, 2001; Lyubomirsky et al., 2011a). Compared with individuals with lower levels of happiness, individuals with higher levels of happiness are more able to maintain and enhance positive emotions in their working memory (Pe et al., 2013), while individuals with lower subjective well-being are more likely to have negative life events and be more sensitive and tend to indulge in self-negative thinking (Kringelbach and Berridge, 2009; Lyubomirsky et al., 2011b). At the same time, individuals who have low subjective well-being and experience negative emotions such as depression and anxiety will show a higher tendency toward smartphone addiction (Demirci et al., 2015; Ahn, 2016). A meta-analysis by Elhai et al. (2017) further supports this conclusion; they pointed out that depression and anxiety are indeed important predictors of individual smartphone addiction.

In general, based on the above-mentioned literature review, we have good reasons to expect that subjective well-being will play a mediating role between self-consistency congruence and smartphone addiction.

### The Moderating Role of Gender

Gender differences in psychology and behavior are a very important field of psychological research (Li and Yang, 2008; Chen et al., 2014, 2020). Many studies have examined the gender differences in smartphone addiction (Ko et al., 2005; Ha and Hwang, 2014). A survey in South Korea found that girls’ smartphone addiction levels are higher than boys’ (Li, 2014), van Deursen et al. (2015) studies have found that women have a higher chance of smartphone addiction. Other studies have found that there are pattern differences between different genders in self-esteem, life satisfaction, frustration tolerance, and smartphone addiction (Ko et al., 2005, 2008). Therefore, we have reason to speculate that gender differences play a moderating role in the influence of adolescents’ self-consistency congruence and subjective well-being on smartphone addiction. Although there is not yet research to test this speculation, there is preliminary support for this regulation in the literature.

For example, studies have shown that as the level of self-consistency congruence rises, the happiness of female college students increases faster than that of male college students (Liu et al., 2014). Another study pointed out that with the increase in positive emotions, women’s smartphone addiction levels declined faster than men’s (Inglehart, 2002). In a study on how self-esteem predicted well-being in college students, Reid (2004) found that male college students’ self-esteem was more predictive of their well-being than it was in female college students. Research on self-esteem, self-efficacy and life satisfaction found, that compared with women, men’s self-esteem and self-efficacy have a stronger predictive effect on life satisfaction (Vecchio et al., 2007).

### The Present Study

**FIGURE 1:**
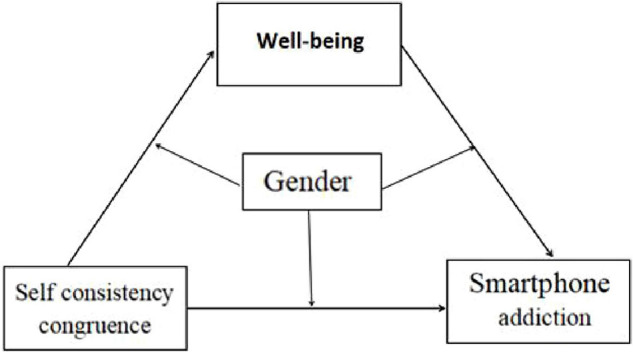
Intermediary model with regulation.

Taken together, the present study investigated whether subjective well-being would act as a mediator between self-consistency congruence and adolescent smartphone addiction, and whether gender would moderate the direct and/or indirect associations between self-consistency congruence and adolescent smartphone addiction (Figure 1).

## Materials and Methods

### Participants and Procedure

Using the overall sampling method, the researchers selected four junior high schools from Jiangxi Province for the questionnaire survey. In total, 1,011 questionnaires were distributed; 946 questionnaires were collected; and 941 valid questionnaires were returned, of which 467 (49.4%) were completed by boys and 473 (50.6%) by girls; 353 (37.3%) of respondents were in the first grade, 332 (35.1%) in the second grade, and 203 (21.5%) in the third grade.

This study was approved by the Research Ethics Committee of Nanchang University. Before data collection, participants were provided with written informed consent forms, which included a brief description of the research project and informed participants that any information provided would be kept confidential with no names recorded, that their responses would be used only for research purposes, and that they were free to discontinue their participation at any time without penalty. The participants were then invited to complete all questionnaires, which were distributed in the same order. The measures were administered, and brief biographical data were collected by well-trained postgraduate students. The participants took approximately 25 min to complete the questionnaires.

### Measures

#### Self-Consistency Congruence Scale

The self-consistency congruence scale was designed by Wang (1994) based on the seven dimensions proposed by Rogers and was developed from the therapist’s own assessment to the subject’s self-assessment. The scale was divided into three dimensions: “Dissonance between self and experience” (16 items), “Flexibility of self” (12 items), and “Stereotypicality of self” (7 items). Items were rated on a scale of 1-5 from “not at all” to “completely.” The scale was reverse scoring, whereby the scores of the items included in each dimension were added up to the factor scores, and the score of self-flexibility was reversed, while the sum of the three factors was the total score of self-consistency congruence. The total score ranged from 35 to 175, with higher scores indicating lower levels of self-concordance. The internal consistency coefficient of this scale was 0.85, and the factors were 0.50−0.76. The Pearson correlations between the factors and with the total scale were 0.40−0.49 and 0.62−0.73, respectively. The scale has good reliability and validity in many studies (Wang and Cui, 2006, 2007).

#### Smartphone Addiction Index

The Smartphone Addiction Index (SAI) was revised by Huang et al. (2013). It consisted of 17 entries divided into four dimensions: loss of control, withdrawal, avoidance, and ineffectiveness. Each entry was scored using a 5-point scale, with “never,” “occasionally,” “sometimes,” “often” and “always,” the higher the total score, the higher was the degree of smartphone dependence. The Cronbach’s alpha coefficient of this study’s questionnaire was 0.90 (Huang et al., 2013). The scale has good reliability and validity in many studies (Hou et al., 2021; Liu et al., 2021).

#### General Well-Being Schedule

The General Well-Being Schedule (GWB), revised by Duan et al. was used to measure individuals’ subjective well-being (Duan, 1996), with 18 entries divided into six dimensions: worry about health, energy, satisfaction and interest in life, depressed or pleasant state of mind, control over emotions and behavior, and relaxation and tension. A Likert-type scale was used, with higher scores associated with higher subjective well-being. The Cronbach’s alpha coefficient of the questionnaire in this study was 0.88 (Duan, 1996). The scale has good reliability and validity in many studies (Chu et al., 2021; Xin et al., 2021).

### Date Analysis

SPSS 22.0 and SPSS macro-PROCESS were used to analyze the data (Hayes, 2013). Descriptive statistics were first examined, followed by computing Pearson’s correlation coefficients to assess the associations among the variables. Finally, PROCESS (Model 6) was applied to test the multiple-mediation models that involved self-consistency congruence and smartphone addiction with the association of subjective well-being and gender.

## Results

### Common Method Deviation Test

The Harman one-factor test was used to test for common method bias. The results showed 18 factors with characteristic roots greater than 1, explaining 54.88% of the total variance, and the amount of variance explained by the first factor was 16.24%, based on L. R. Long’s criterion (Zhou and Long, 2004), when the critical criterion was < 40%, indicating no serious common method bias variance.

### Primary Analysis

The mean, standard deviation, and correlation coefficient matrices for all variables are presented in Table 1. Self-consistency congruence scores (the higher the score, the lower the harmony) were significantly negatively correlated with well-being; well-being was negatively correlated with cellphone addiction; and self-consistency congruence scores were positively correlated with smartphone addiction.

**TABLE 1:** Correlation analysis of variables.

Variables	*M*	*SD*	1	2	3	4
Gender			1			
Self-consistency congruence	116.93	12.67	0.069*	1		
Well-being	87.00	10.90	−0.044	−0.375**	1	
Smartphone Addiction	36.98	14.22	−0.028	0.412**	−0.393**	1

*Indicates significant.

**Indicates extremely significant.

### Intermediary Model Test With Moderation

First, to test the mediating role of well-being between self-consistency congruence and adolescent smartphone addiction, a mediation effect analysis was conducted using Model 4 in the PROCESS program developed by Hayes. The results showed that self-consistency congruence scores positively predicted adolescent smartphone addiction (β = −0.3226, *t* = −12.4302, *p* < 0.01) and negatively predicted well-being (β = −0.3454, *t* = 10.0251, *p* < 0.01), and well-being negatively predicted smartphone addiction (β = −0.3614, *t* = −9.0222, *p* < 0.01). Bootstrap 95% confidence intervals for both the direct effect of self-concordance on smartphone addiction and the mediating effect of well-being did not contain 0 (see Table 2), indicating that well-being partially mediates the effect between self-concordance and smartphone addiction. This direct effect (0.3454) and mediating effect (0.1166) accounted for 74.8 and 25.2% of the total effect (0.4620), respectively.

**TABLE 2:** Mediating role of well-being.

Smartphone addiction	Effect value	Boot standard error	Boot CI lower limit	Boot CI upper limit	Relative effect value
Total effect	0.4620	0.0333	0.3967	0.5273	
Direct effect	0.3454	0.0345	0.2778	0.4131	74.8%
The mediating effect of well-being	0.1166	0.0177	0.0841	0.1534	25.2%

Next, the mediated model with moderation was analyzed using Model 59, and the results are presented in Table 3. After gender was entered into the model, the self-consistency congruence score significantly and negatively predicted well-being (β = −0.3196, *t* = −12.2865, *p* < 0.01), and gender was not a significant predictor of well-being (β = −0.3971, *t* = −0.6027, *p* > 0.05). The interaction term of the self-consistency congruence score and gender was a significantly negative predictor of well-being (β = −0.1135, *t* = −2.1814, *p* < 0.05); the self-consistency congruence score was a significantly positive predictor of smartphone addiction (β = 0.3443, *t* = 9.994, *p* < 0.01); gender was a negative predictor of smartphone addiction (β = −1.7609, *t* = −2.178, *p* < 0.05); and well-being was also a significant predictor of smartphone addiction (β = −0.3629, *t* = −9.0732, *p* < 0.01). The interaction item of the self-consistency congruence score and gender was not a significant predictor of smartphone addiction (β = −0.0759, *t* = −1.1011, *p* > 0.05); the interaction item of well-being and gender was a significant predictor of smartphone addiction (β = −0.0759, *t* = −1.1011, *p* > 0.05); and the interaction item of well-being and gender was a significant predictor of smartphone addiction. The interaction term of well-being and gender was a significantly negative predictor of well-being (β = 0.2106, *t* = −2.6321, *p* < 0.01).

**TABLE 3:** Intermediary model with moderating.

	Regression equations	Overall fit index			Significance of regression coefficients	
			
Result variables	Predictive variables	R	R2	F	β	*t*
Well-being		0.3811	0.1453	53.3104**		
	Self-consistency congruence(a)				–0.3196	−12.2865**
	Gender(b)				–0.3971	–0.6027
	a × b				–0.1135	−2.1814*
Smartphone addiction		0.4951	0.2452	60.9982**		
	Self-consistency congruence(a)				0.3443	9.994**
	Gender(b)				–1.7609	−2.178*
	Well-being(c)				–0.3629	−9.0732**
	a × b				–0.0759	–1.1011
	c × b				–0.2106	−2.6321**

*Indicates significant.

**Indicates extremely significant.

The direct-effect values for self-concordance, mediated effect values for well-being between self-concordance and smartphone addiction, and Bootstrap 95% confidence intervals for both male and female conditions are shown in Table 4.

**TABLE 4:** Direct and mediating effects under different gender conditions.

Smartphone addiction	Gender	Effect value	Boot standard error	Boot CI lower limit	Boot CI upper limit
The direct effect of self-consistency congruence	Male	0.4498	0.0491	0.2862	0.479
	Female	0.3068	0.0484	0.2117	0.4018
The mediating effect of well-being	Male	0.0672	0.0198	0.0324	0.1089
	Female	0.1754	0.0285	0.1218	0.2336

Further, a simple slope analysis was performed. The negative predictive effect of adolescent boys’ self-consistency congruence scores on well-being was significant (β = −0.2622, *t* = −6.9613, *p* < 0.01), and the negative predictive effect of adolescent girls’ self-consistency congruence scores on well-being was significant (β = −0.3757, *t* = −10.4573, *p* < 0.01), but compared to boys, as self-consistency congruence scores increased, girls’ well-being declined more rapidly. The negative predictive effect of adolescent well-being on smartphone addiction was significant (β = −0.3948, *t* = −6.9176, *p* < 0.01), and the negative predictive effect of adolescent girls’ well-being on smartphone addiction was significant (β = −0.6189, *t* = −11.6298, *p* < 0.01), but compared to boys, as their well-being increased, girls’ smartphone addiction scores declined more rapidly.

## Discussion

### The Effect of Self-Consistency Congruence on Adolescent Smartphone Addiction

The findings showed that self-consistency congruence significantly and negatively predicted adolescent smartphone addiction, and the higher the self-consistency congruence, the lower the probability of adolescent smartphone addiction. Self-stereotypy refers to how individuals perceive themselves as possessing attributes associated with a group when they identify with the group. Self-flexibility refers to an individual’s smart adaptation to a changing situation and environmental requirements; this is an important psychological resource that allows individuals to cope with unfavorable situations. In these results, self-inconsistency with experience and self-stereotypy both positively predicted smartphone addiction in adolescents, and self-flexibility did not predict smartphone addiction in adolescents, which is consistent with the findings of Liu and Yao’s (2006) study on the relationship between self-consistency congruence and smartphone addiction in college students. Adolescents are in the critical period of self-unity formation (Wang et al., 2011). Their self-concept is prone to deviations and instability, with frequent inconsistencies between self-concept and external experience, and adolescents often use smartphone addiction as a means to escape from self-contradictions and conflicts. Adolescents with stereotyped egos have low levels of psychological resilience and are often overwhelmed when they encounter new situations and problems. The temporary pleasure smartphone use brings them at this time can alleviate this sense of panic, and although they may regret it afterward, they cannot help but continue to rely on their smartphones to relieve their emotions once they encounter insurmountable difficulties. One study found that the alcohol dependence behavior of subjects in the self-loss group was significantly higher than that of the non-self-loss group (Leith and Baumeister, 1996), which shows that self-loss can easily lead to dependence behavior. Subjects with low self-concordance naturally have high self-loss, which then tends to lead to smartphone-addictive behaviors. Therefore, low self-consistency congruence is an important predisposing factor for adolescent internet-addictive behaviors.

### Analysis of the Mediating Effect of Well-Being

Well-being is a psychological state created by an individual’s satisfaction with their mental condition and an overall evaluation of the positive and negative emotions experienced. Positive emotions are experienced when the individual is satisfied to a high degree, and negative emotions are experienced when the individual perceives a low degree of satisfaction. Negative emotions are emotional dimensions that are influenced by internal and external factors that have a negative impact on an individual’s physical and mental development and social functioning, and the most common negative emotions are anxiety, anger, fear, low self-esteem, and resentment (Plutchik, 1982). The present study found that well-being negatively predicted adolescents’ internet-addictive behaviors. When individuals’ well-being was reduced, they experienced strong negative emotions, and their negative emotions were able to positively predict their smartphone addiction behaviors (Li D. et al., 2016). This is likely because when adolescents have a low sense of well-being, they often experience negative emotions and suffer from mental distress. According to the compensation theory of smartphone addiction, smartphone-addictive behavior is a psychological compensation behavior adopted by individuals when they suffer from mental distress, so well-being can negatively predict adolescents’ smartphone-addictive behavior.

Self-consistency congruence refers to the coherence of the internal parts of the self as well as the coherence of the inner self with external experiences. Individuals with high self-concordance often experience ease and pleasure because of the coherence of the internal parts of the self and the harmony between the self and external experience. However, individuals with low self-concordance often experience negative emotions because parts of their ego and external experiences are in conflict, leading to a decrease in their sense of well-being. The present study found that self-consistency congruence positively predicted adolescent well-being and that when low self-consistency congruence was accompanied by low levels of well-being among adolescents, they were prone to experience internal and external conflicts. This is consistent with the findings of a study conducted by Li and Wu, 2001. Satir’s (2015) internal iceberg theory also suggests that self-consistency congruence at the deepest level influences well-being at the middle level and, in turn, affects cellphone-addictive behaviors as the outermost level. Therefore, parents and society should pay attention to adolescent psychological harmony and help adolescents to achieve self-growth, thus enhancing their well-being and reducing adolescent internet-addictive behaviors.

### Analysis of the Moderating Effect of Gender

Gender was found to play a moderating role in the relationship between self-concordance and well-being in adolescents. Adolescent girls experience a more rapid decline in well-being than boys as their self-consistency congruence decreases because females are more emotionally sensitive and perceptive than males (Yuan et al., 2010), and when girls face internal and external self-conflict and lower self-consistency congruence, they feel more intensely negative emotional experiences than boys, and their well-being declines more rapidly. This suggests that girls with low self-consistency congruence should receive more attention, support, and protection in the process of youth mental health education and maintenance. However, the increased attention to girls does not mean that the influence of adolescent boys’ self-consistency congruence on well-being can be ignored, because males are only used to rational thinking and expressing emotions introvertedly, and often tend to hide their emotions (Li and Du, 2015). Thus, we need to pay equal attention to the education and diversion of negative emotions implicit in adolescent boys with low self-consistency congruence.

The study also found that gender plays a moderating role in the relationship between adolescent well-being and smartphone addiction. As well-being increases, youth smartphone addiction decreases, but girls experience a faster decline than boys, which is consistent with the findings of Lee (2014). Girls are more emotionally delicate and have stronger emotional needs, and once these needs are not met, for example when they experience the negative emotional experience of unwell-being, they are more likely to resort to smartphone addiction as a way to relieve their negative emotions. In contrast, boys are more likely to be addicted to online games, and although they can also play games on smartphones, they are more likely to prefer large competitive games on PCs (computer clients) (Ding et al., 2018). Therefore, as adolescent boys’ well-being decreases, their negative emotions increase more slowly than those of girls as smartphone dependence increases.

## Limitations and Future Research

While we have conducted an extended study of the relationship between self-consistency congruence and smartphone addiction, some shortcomings remain.

First, although our study was helpful in understanding antecedents to smartphone addiction, causal relationships cannot be supported, as our study employed a cross-sectional design. Follow-up studies that incorporate experimental or longitudinal designs have greater potential to identify any possible cause-and-effect relationships among these variables.

Second, although the participants in this study were enrolled in four ordinary middle schools in China, they may not have fully represented all the teenagers in China. Thus, the extent to which our findings can be generalized to other populations cannot be determined.

We chose to focus intensively on internal factors, such as self-consistency congruence and subjective well-being, but we acknowledge that various external or environmental factors may play important roles as well. Simultaneous examination of individual and environmental factors, drawing upon multiple sources of data, may lead to further insights into the key determinants of smartphone addiction and how to address it and minimize its adverse impact.

Finally, the relationship between self-consistency congruence and internal indicators of subjective well-being can be tested in future research, such as positive emotions, negative emotions, and life satisfaction. These indicators are not included in the current research, and future research can be further explored.

## Conclusion

The present study was designed to explore a new perspective by identifying other important mediators between self-consistency congruence and smartphone addiction among adolescents. We found that subjective well-being mediates the relationship between these two variables. Of these, subjective well-being played a partial mediator role, but gender moderated the association between self-consistency congruence and smartphone addiction, compared with boys, girls’ self-consistency congruence changes are more likely to cause changes in smartphone addiction.

This result suggests that the adolescents’ smartphone addiction may be related to self-consistency congruence and subjective well-being, and there are gender differences in this relationship. This research provides ideas for interventions on adolescents’ smartphone addiction from the perspective of self-consistency congruence and subjective well-being.

## Data Availability Statement

The original contributions presented in the study are included in the article/supplementary material, further inquiries can be directed to the corresponding author/s.

## Ethics Statement

The studies involving human participants were reviewed and approved by Academic Committee of Nanchang University. Written informed consent to participate in this study was provided by the participants’ legal guardian/next of kin.

## Author Contributions

YL: methodology, formal analysis, and writing—original draft. XM: writing—review and editing and investigation. CL: translation and polishing. CG: revised, validation, and supervision. All authors have read and agreed to the published version of the manuscript.

## Conflict of Interest

The authors declare that the research was conducted in the absence of any commercial or financial relationships that could be construed as a potential conflict of interest.

## Publisher’s Note

All claims expressed in this article are solely those of the authors and do not necessarily represent those of their affiliated organizations, or those of the publisher, the editors and the reviewers. Any product that may be evaluated in this article, or claim that may be made by its manufacturer, is not guaranteed or endorsed by the publisher.
